# Thermodynamic reasoning for colossal N supersaturation in austenitic and ferritic stainless steels during low-temperature nitridation

**DOI:** 10.1038/s41598-019-44410-0

**Published:** 2019-05-29

**Authors:** K. N. Sasidhar, S. R. Meka

**Affiliations:** 1 Department of Metallurgical and Materials Engineering, Indian Institute of Technology Roorkee, Roorkee, 247667 India; 2 Department Microstructure Physics and Alloy Design, Max-Planck-Institut für Eisenforschung GmbH, Düsseldorf, Germany

**Keywords:** Chemical physics, Computational methods

## Abstract

Colossal N supersaturation of ferritic as well as austenitic stainless steels during low temperature gaseous nitridation treatments has lately gained much technological significance. However, available thermodynamic models to calculate the N paraequilibrium solubility limits have failed to explain the levels of colossal N supersaturation observed in several cases of nitrided ferritic/austenitic stainless steels. In this work, we show that consideration of N dissolution induced spinodal decomposition is essential in calculating the N paraequilibrium solubility limit for both ferritic and austenitic stainless steels. This modification in the thermodynamic model has led to the successful explanation of the thermodynamic cause for the colossal N supersaturation in ferritic and austenitic stainless steels. Available experimental observations in literature support the occurrence of spinodal decomposition.

## Introduction

Surface modification of stainless steels for the purpose of simultaneously achieving improved corrosion resistance and surface mechanical properties is of great technological significance^1–4^. In this context, application of nitriding treatments to surface harden the stainless steels has always been a challenging task due to the strong chemical affinity of Cr to N which is known to result in the formation of Cr-nitrides. Formation of these Cr-nitrides deteriorates the corrosion resistance of nitrided stainless steel components due to removal of Cr from the solid solution (necessary for corrosion resistance). Because of this reason the application of nitridation treatments to stainless steels was traditionally not possible, although the nitridation treatments were widely employed for improving the mechanical and chemical properties of surfaces of non-stainless steel components^5,6^. The application of nitriding treatments to stainless steels has become common in industry only after the invention of the low temperature nitriding treatments which upon optimized application lead to the development of nitrided layers with only colossal amounts of N dissolved in ferrite/austenite without any Cr-nitride precipitation^7–9^. Implementation of this approach has led to a great minimization of the problem of corrosion resistance deterioration of stainless steels after nitriding and in certain situations the corrosion resistance has even been enhanced^7,10,11^. Although the solid solution hardening is not as effective as precipitation hardening, colossal amounts of N dissolved in ferrite/austenite solid solutions of low temperature nitrided stainless steels have resulted in hardness levels of treated stainless steel surfaces comparable to those achieved by the usual precipitation hardening mechanism. Due to this, significant developments have taken place in producing colossal N supersaturation in stainless steels while suppressing the Cr-nitrides development^7,9,11^.

Because of the importance of understanding and optimizing the colossal levels of N dissolution, researchers have made attempts to calculate the maximum N paraequilibrium solubility which can be realized for applied chemical potential of N (which depends on T and nitriding potential employed)^12^ in nitriding atmosphere for different ferritic and austenitic stainless steels while suppressing Cr-nitrides development^7,11,13,14^. This limit is defined by the point where the thermodynamic driving force for absorption of nitrogen into the ferritic or austenitic solid solution from nitriding atmosphere vanishes, i.e. when chemical potential of atomic nitrogen in the ferritic/austenitic solid solution (in the work piece) becomes equal to that constantly maintained in the nitriding atmosphere. Such calculations for the case of controlled gaseous nitriding of stainless steels, which allow precise control of chemical potential of N in gas phase, are expected to correctly predict the measured nitrogen contents.

Although such thermodynamic model calculations appear theoretically correct, until now such calculations have failed to explain the measured levels of N supersaturations realized in stainless steels; the calculated N paraequilibrium solubility limits for AISI 316 austenitic stainless steel was much greater than the observed N contents (see Fig. 7 in^12^), whereas the calculated paraequilibrium N content for delta-ferrite of 17-7 PH stainless steel is much smaller than the observed content^11,15^. Such N absorption, not compatible with the theoretical limits is thermodynamically unreasonable. These observations clearly show that the currently available thermodynamic models are inadequate in describing the low temperature nitridation process. A recent work^16^ has shown the possibility for occurrence of spinodal decomposition during nitridation of ferritic Fe-Cr alloys under conditions of restricted substitutional element diffusion, influencing the phase transformation pathway chosen by the system during nitriding. In this work, we show that a similar phenomenon occurs also during nitridation of ferritic and austenitic stainless steels. We specifically show that consideration of this, until now unrecognized phenomenon of *in-situ* spinodal decomposition during nitridation of ferritic or austenitic stainless steels, in calculating the N paraequilibrium solubility limit leads to a successful explanation of the reported experimental observations. It is interesting to note that significant evidence exists in literature for the occurrence of spinodal decomposition in delta-ferrite of austenitic stainless steels upon thermal aging^17^. Yet, the role of spinodal decomposition in determining the N paraequilibrium solubility limit in nitrided austenitic and ferritic stainless steels (nitrided at temperatures similar to that of the thermal aging treatments) has until now not been recognized.

## Thermodynamic Calculations

### Classical N paraequilibrium solubility limit and the need for its modification

Thermodynamic calculations have been carried out, firstly to find the minimum N supersaturation levels in ferritic and austenitic stainless steels required to create the spinodal instability in the respective N supersaturated solid solutions. This spinodal limit for multi-component solid solutions is obtained by evaluating the Hessian matrix of the Gibbs free energy function (*G*) of the respective solid solutions. The mathematical condition for obtaining the spinodal limit can be written as follows^18^:

1 $$|\begin{array}{llll}{g}_{22} & \cdot  & \cdot  & {g}_{2c}\\ : & : & : & :\\ {g}_{c2} & \cdot  & \cdot  & {g}_{cc}\end{array}|=0$$

Where, *c* is the number of components in the solid solution and ${g}_{kl}(=\frac{{\partial }^{2}G}{\partial {x}_{l}\partial {x}_{k}})$ is the second derivative of the Gibbs free energy function ${G}_{m}^{solid}$ of the respective solid solutions with respect to the independent composition variables (which are (*c* − 1) in number).

Calculations have been carried out for the chemistry of the austenitic AISI 316 stainless steel (utilized in^7^) and the chemistry of delta-ferrite of 17-7 PH stainless steel (utilized in^11^) (Table 1) as a function of temperature.

**Table 1:** Chemical compositions of the stainless steel grades employed in^7^ and^11^ respectively.

Element	Cr	Ni	Mo	Al	Mn	Si
Austenite of AISI 316 SS^7^	19.1	12.7	1.4	—	1.66	1.26
Delta-ferrite of 17-7 PH SS^11^	22	4	—	2.5	0.6	1

After that the N paraequilibrium solubility limits for the same un-decomposed austenitic and ferritic solid solutions have been respectively calculated, as a function of temperature for a particular applied nitriding potential of 0.6 atm^−1/2^ (employed in^11^). The N-paraequilibrium solubility limit is the point when the chemical potential of nitrogen in the solid solution becomes equal to that of the gas phase, and thus indicates the saturated N-content absorbed by the specimen under the given nitriding conditions. This condition can be written as

2 $${\mu }_{N}^{gas}=\frac{1}{2}{G}_{{N}_{2}}^{0}+RTln{a}_{N}^{gas}={\mu }_{N}^{solid}={\mu }_{N}^{0}+RTln{a}_{N}^{solid}$$

${G}_{{N}_{2}}^{0}$ is the Gibbs free energy of pure nitrogen gas at the nitriding temperature and pressure. The N activity in the gas phase ${a}_{N}^{gas}$ is obtained by

3 $${a}_{N}^{gas}=k\frac{{p}_{N{H}_{3}}}{{p}_{{H}_{2}}^{3/2}}=k{r}_{N}$$

Where *k* is the equilibrium constant of the ammonia decomposition reaction and *p*_*i*_ represents the partial pressure of the species ‘*i*’. ${r}_{N}(=\frac{{p}_{N{H}_{3}}}{{p}_{{H}_{2}}^{3/2}})$ is defined as the nitriding potential^5^. Nitriding potential, as can be seen from (2) and (3) determines the chemical potential of N in the gas phase ${\mu }_{N}^{gas}$. The N-content in the respective homogeneous solid solutions corresponding to this chemical potential/activity of N is obtained by finding the solid solution composition point at which the line tangent to the vertical section of the respective Gibbs free energy functions along the direction of nitriding (in the compositional space), makes an intercept of ${\mu }_{N}^{gas}$ on the 100% N axis in the compositional space (for further details on the geometrical construction, please see^16^). This is the N solubility limit expected upon suppressing the spinodal decomposition and all other Cr-nitrides development. The Gibbs free energies $({G}_{m}^{solid})$ of the ferrite or austenite solid solutions of the respective stainless steels have been derived by extending the Hillert-Staffanson sub-lattice model (the standard CALPHAD approach)^19^, in accordance with the methodology employed by Wu *et al*.^12^.

**Figure 1:**
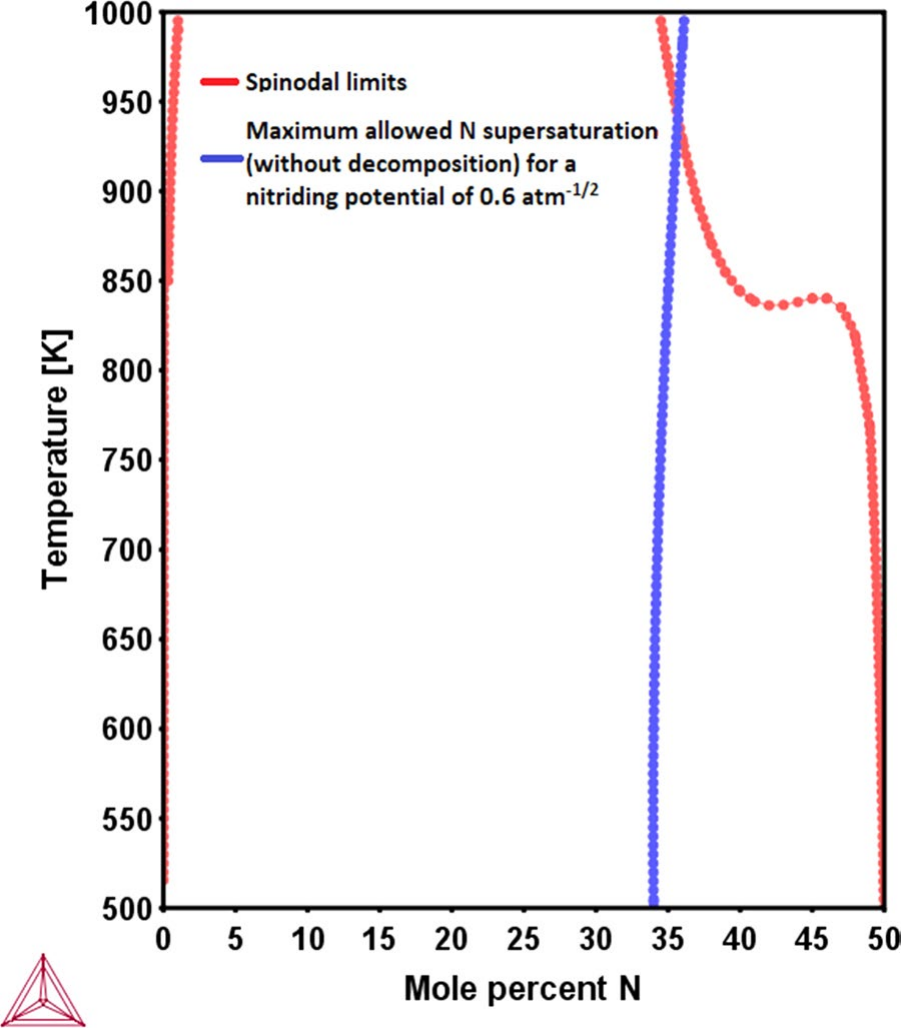
Spinodal limit for the austenitic solid solution of AISI 316 steel (Fe-19Cr-13Ni-1.5Mo-1.6Mn-1.3Si (at %)) as a function of temperature and N content (red curve). The paraequilibrium N solubility limit of austenite as a function of temperature for a nitriding potential of 0.6 atm^−1/2^ (blue curve).

**Figure 2:**
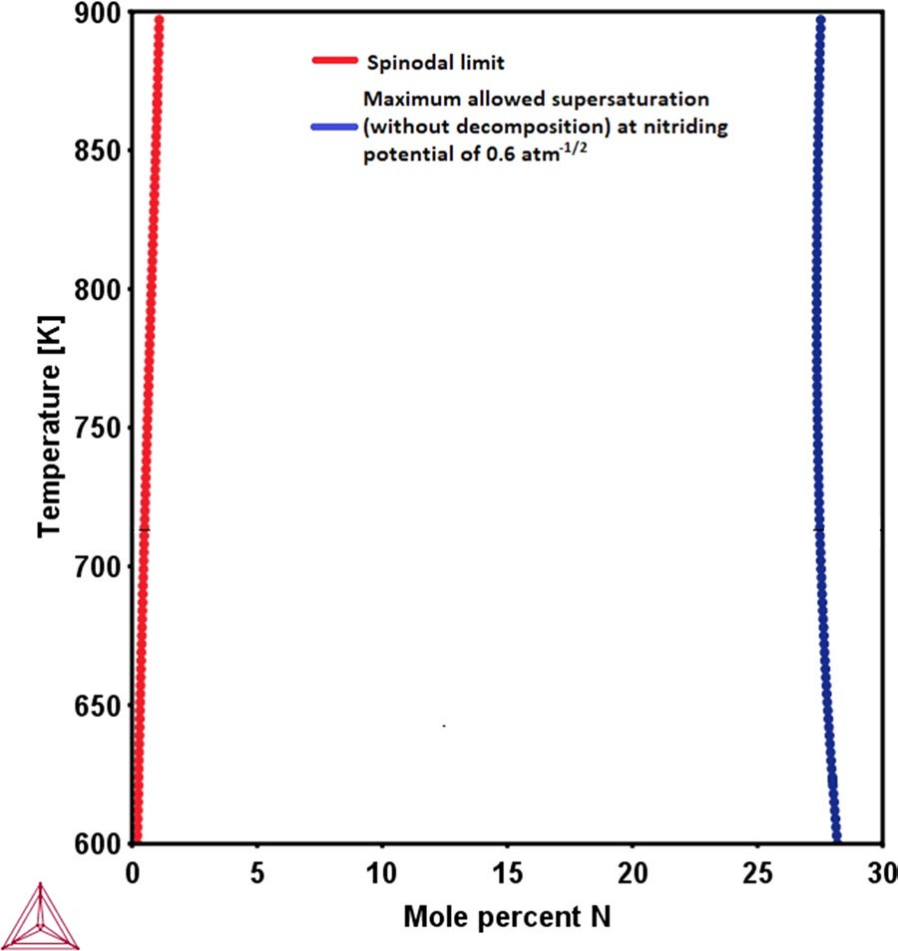
Spinodal limit for the delta ferritic solid solution (Fe-22Cr-4Ni-2.5Al (in at %)) of stainless steel as a function of temperature and N content (red curve). The paraequilibrium N solubility limit of delta-ferrite as a function of temperature for a nitriding potential of 0.6 atm^−1/2^ (blue curve).

These calculations (of the spinodal limits and the N-paraequilibrium solubility limits) have been performed with the help of Thermo-Calc ver. 2017 (b) Software. Thermodynamic data for calculations has been obtained from TCFE9 database which is based on the thermodynamic data presented in the literature)^20–25^. The thus calculated results have been presented in Figs 1 and 2. The results obtained clearly indicate that the minimum N amount required to bring the spinodal instability in the ferritic as well as austenitic solutions is much smaller than the paraequilibrium N solubility limit allowed for the austenitic (AISI 316 steel) and delta-ferritic(17-7 PH steel) solutions. This means that in the course of nitridation, the developing N supersaturated ferritic and austenitic solid solutions of stainless steel, sample regions in the compositional space falling within the region of spinodal instability. Therefore, the austenite and delta ferrite solid solutions, which are stable to spinodal fluctuations prior to nitriding, become prone to spinodal decomposition in the course of N-absorption *in-situ* during nitriding of AISI 316 and 17-7 PH stainless steels, respectively.

In other words, from the moment the N content reaches the level which triggers the spinodal decomposition (red curves in Figs 1 and 2) during nitriding, the composition pathway traversed by the respective austenitic and ferritic solid solutions in the isothermal composition space changes; depending on the ease of achieving a stable spinodal compositional fluctuation. This means that the N compositions corresponding to the above calculated N-paraequilibrium solubility limits (red curves in Figs 1 and 2) *would never be sampled by the single, homogeneous solid solutions*. Rather, phase separation begins at N contents corresponding to the spinodal limits. In the context of these findings, the concept of considering a homogeneous N-supersaturated ferritic or austenitic solid solution to be in paraequilibrium with the nitriding atmosphere is fundamentally flawed. Therefore, a realistic description of the final paraequilibrium state of the ferritic or austenitic solid solutions, respectively with the gas atmosphere (in order to obtain the N-paraequilibrium solubility limit) requires a modification. The details of this modified paraequilibrium are presented below.

### Modified N paraequilibrium solubility limit for spinodally decomposed austenite/ferrite solid solutions

**Figure 3:**
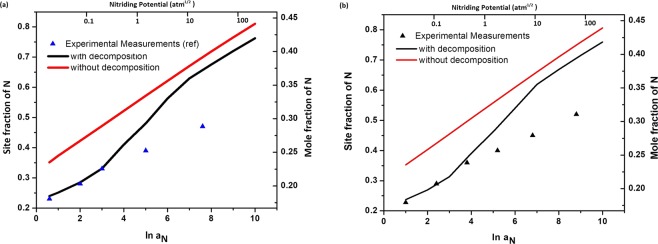
Isothermal N paraequilibrium solubility limits calculated for austenitic solid solution of AISI 316 stainless steel by the method proposed in this work (i.e. with consideration of N-induced spinodal decomposition, black curve) for different N activities in the gas phase (as considered in^7^) or nitriding potentials. The measured levels of N supersaturation (from^7^, triangles) and N paraequilibrium solubility limits calculated without consideration of spinodal decomposition (using method used in^12^, red curve) have also been indicated (**a**) at 693 K and (**b**) at 718 K.

**Figure 4:**
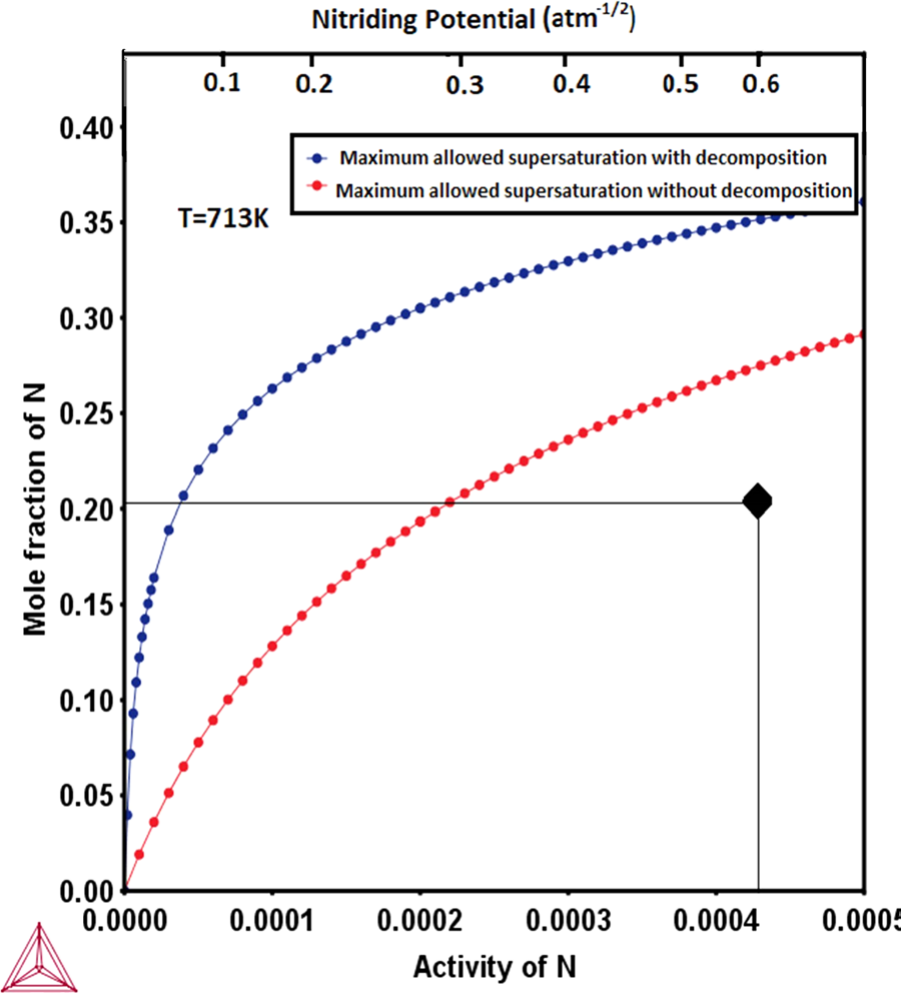
N paraequilibrium solubility limit of delta-ferrite at nitriding temperature of 713 K, as a function of nitriding potential/N activity (with respect to standard element reference (SER)) calculated by the method proposed in this work (blue curve) and by the method used in^12^. The measured N content (in^11^) is also indicated.

Since phase separations has been shown (from Figs 1 and 2) to be feasible at relatively very low N-contents than those observed, the paraequilibrium that is closer to reality is that between the spinodally decomposed (phase separated) solid solutions and the nitriding atmosphere rather than between the un-decomposed, homogeneous solid solution and nitriding atmosphere. This kind of chemical equilibration involving two distinct solid solution compositions pertaining to the same phase (austenite or ferrite, respectively) with the gas atmosphere was achieved simply by imposing the condition of global minimization of Gibbs free energy of the solid solution mixture while imposing the condition that the chemical potential of N in all solid solution phases is equal to that in the gas phase. The limit of this modified N paraequilibrium contents calculated for both the austenitic AISI 316 stainless steel and the delta-ferrite of the 17-7 PH stainless steel are presented in Figs 3 and 4, as a function of different nitrogen activities in the nitriding atmosphere along with the calculated N paraequilibrium values for un-decomposed austenite and ferrite solid solutions and experimentally measured values from literature.

Before discussing the calculated results, it must be mentioned herein that the validity of these calculations hinges on the validity of extrapolating the thermodynamic data of ferritic and austenitic solid solutions (cited above) measured and assessed at relatively low N contents into the metastable, N-supersaturated states considered in this work. As will be shown in the discussion, the close correlation between the calculated results and experimental observations in literature suggests that such extrapolations carried out in this work are quite reasonable.

## Discussion

With the aim of measuring the thermodynamic activities of N dissolved in the colossally supersaturated austenite, or the so-called expanded austenite, Christiansen and Somers^7^ carried out controlled gaseous nitriding experiments on thin foils of AISI 316 stainless steel to realize the complete equilibration of sample with gas atmosphere, i.e. up to the point where no more N is dissolved upon further exposure to the nitriding atmosphere. Only such dedicated experiments allow one to determine the saturation/equilibrium level of N taken up by the sample. It can be seen in Fig. 3 that the N paraequilibrium solubility limits predicted by our method which considers the spinodal decomposition/phase separation are much closer to these experimentally observed equilibrium levels of N supersaturation as compared to the significantly higher values predicted by the previously employed method^12^. In fact, the experimental values fit exactly with our predictions up to a gaseous nitrogen activity of about e^4^ (which is already a very high nitriding potential). However, beyond this N activity in the gas phase, the paraequilibrium N solubility limit is overestimated even by our method. It is known that at high nitriding potentials, the rate of the de-nitriding reaction, i.e. formation of gaseous N_2_ at the specimen surface from N dissolved in the solid begins to compete with the rate of the nitriding reaction^6,26^. The nitriding and de-nitriding reactions are respectively given as follows

4 $$\begin{array}{cc}2N{H}_{3(g)}\to 2{[N]}_{solid}+3{H}_{2(g)} & {k}_{1}\end{array}$$

5 $$\begin{array}{cc}2{[N]}_{solid}\to {N}_{2(g)} & {k}_{2}\end{array}$$

[*N*_*solid*_] refers to atomic nitrogen dissolved in the solid and *k*_1_ and *k*_2_ are the respective rate constants for the given reactions.

Thus, increase in concentration of dissolved N increases rate of reaction (5) . Consequently, the ratio of actual NH_3_/H_2_ partial pressures in the nitriding atmospheres becomes smaller than that expected from the imposed NH_3_/H_2_ flow rates leading to the establishment of an effective gaseous N activity smaller than expected. This effect can be quantitatively corrected, with the help of experimentally determined rate constants *k*_1_ and *k*_2_ of the nitriding and de-nitriding processes^6,26^. However, due to the lack of availability of this experimental data for the stainless steels, it has not been carried out in this work. Nevertheless, it can be seen that the proposed model is very much accurate for lower values of gaseous N activities.

One reason why such a thermodynamic methodology has not been considered in literature previously is the popular notion that the expanded austenite is simply an N supersaturated FCC phase, with a homogeneous distribution of N atoms^27^. This was so considered because of the fact that X-ray diffraction studies of these specimens did not result in the detection of any other equilibrium phases such as chromium nitrides^28,29^. However, some recent X-ray spectroscopy^30^ and atom probe tomography^31^ studies have distinctly shown the existence of nano-sized Cr/N enriched regions, finely distributed throughout the austenite matrix. The austenite was still termed as ‘XRD homogeneous’ due to inability of the technique to discern these features due to the possible coherency of these very fine regions with the matrix. The observation of these heterogeneities in the composition of the so-called expanded austenite is naturally expected from the above proposed mechanism of N uptake through spinodal decomposition of the austenite. In fact, the presence of these Cr/N enriched regions was observed even at locations close to the nitrided case/un-nitrided core interface along with at the surface, indicating their formation occurring right from the beginning stages of nitriding^31^. This is also in corroboration with the fact that the N-induced spinodal decomposition is triggered at N contents as low as 0.1 at % at the nitriding temperature of 718 K (Fig. 1). Furthermore, the atom probe and X-ray spectroscopic analysis also indicated that the Cr/N enriched regions are depleted in Ni, which is segregated into the N-depleted austenite matrix^31^.

The directions of the tie lines of the miscibility gap in the compositional space govern the equilibrium composition of the different austenite fractions resulting from spinodal decomposition^32^. These calculations were also performed to estimate the compositions of the equilibrium fractions of the spinodally decomposed austenite at different overall N content in the mixture. They clearly showed Cr enrichment in the N-enriched fraction with enrichment of Ni in the N-depleted fraction, which is in corroboration with all the composition related experimental observations^31^.

In case of the delta-ferrite in 17-7 PH stainless steel, it can be seen that the measured colossal N content^11^ is in fact smaller than even the N paraequilibrium solubility limit calculated herein without consideration of spinodal decomposition (Fig. 4). However, this N content was reported to be much higher than the N paraequilibrium solubility limit by Wang *et al*.^11^ due to most probably not considering the effect of gaseous N activity on the N paraequilibrium solubility limit^15^. Nevertheless, the mechanism of N absorption is actually by spinodal decomposition beyond N contents of about 1 at % (Fig. 2) at 693 K, and therefore the N paraequilibrium solubility limit calculated by consideration of spinodal decomposition is more appropriate in this case as well. Since these treatments by Wang *et al*.^11^ were carried out on bulk specimens (with large cross-section thickness) and that too for 4 hours only, it is not expected that the specimen necessarily reaches the maximum allowed N supersaturation from kinetic considerations. Since the measured N content turns out to be within the calculated N paraequilibrium solubility limit, there is no scientific anomaly in any way. Moreover, it was also observed through HRTEM analysis and atom probe tomography that there exist distinct modulations in composition with very fine nano-sized regions enriched in Cr and N while being depleted in Fe^11^. In a manner similar to the explanation provided for austenitic stainless steel in the previous section, directions of tie lines for the case of the miscibility gap in the delta-ferrite are also in corroboration with these experimental observations. Similar thermodynamic considerations can also explain the colossal C supersaturation in delta-ferrite of 17-7 PH stainless steel during low-temperature carburization^33,34^.

Colossal supersaturation of solutes, much beyond the conventional solubility limit in solid solutions has recently emerged as an aspect of technological interest not only in surface hardening of stainless steels, but also in development of magnetic anisotropy in iron based thin films. Recently, Golden *et al*.^35^ have reported the observation of ferritic iron supersaturated with interstitial Boron to a colossal 14 at% of Boron obtained during thin film growth by molecular beam epitaxy (MBE) at temperatures of about 300 °C. Theoretical calculations predict that precise optimization of interstitial element supersaturations of this level in the iron based thin films can result in favourable magnetic properties^35–37^. The low temperature process of MBE is in fact analogous to the process of low temperature nitridation in the specific sense that it also involves imposition of a particular controlled chemical potential of the elements in the vapour phase, which control the composition of growing thin film. Under the circumstances, it can be argued that the concepts of the paraequilibrium interstitial element solubility discussed in this work, could be applicable for the case of the iron based thin films as well. Therefore, insights gained in this work into the influence of the nature of miscibility gaps in the solid solutions (including the directions of tie-lines) on the maximum allowed interstitial element supersaturation could very well be useful in design and optimization of the elemental composition of these thin films.

## Conclusions

1. The existence of spinodal instability in metastable N supersaturated austenite of AISI 316 stainless steel and the delta-ferrite of 17-7 PH stainless steel has been recognized by thermodynamic calculations. The typically employed chemical potentials of N in the gaseous nitriding atmosphere during low temperature nitridation of stainless steels have been shown to bring the evolving solid solution chemistry into the region of spinodal instability in course of establishing an N paraequilibrium with the nitriding atmosphere. Accordingly the ferritic and austenitic solid solutions of stainless steels undergo spinodal decomposition during nitriding. Evidence for the expected compositional heterogeneities as a result of spinodal decomposition in the expanded austenite and ferrite are in fact available in literature.
2. A new method for determination of the paraequilibrium level of N which can be taken up by the stainless steels upon establishment of the N paraequilibrium between spinodally decomposed ferritic and austenitic solutions with the gas atmosphere has been proposed.
3. For the first time the thermodynamic basis for the observed colossal N supersaturations in low temperature nitrided austenitic AISI 316 stainless steel and the delta-ferrite of 17-7 PH stainless steels has been presented through consideration of spinodal decomposition of the respective solid solutions.
